# Anesthesia inhalation agent sevoflurane induces perioperative cognitive impairment: Apelin/early growth response 1 axis induces ferritinophagy and exacerbates ferroptosis

**DOI:** 10.4103/mgr.MEDGASRES-D-25-00173

**Published:** 2026-09-12

**Authors:** Xiufeng Li, Xiaoying Zhang, Longhe Xu, Yongzhe Liu

**Affiliations:** 1 Department of Anesthesiology, Shanxi Medical University, Taiyuan, Shanxi Province, China; 2 Department of Anesthesiology, the Third Medical Center of Chinese PLA General Hospital, Beijing, China; 3 Department of Anesthesiology, the First Medical Center of Chinese PLA General Hospital, Beijing, China

**Keywords:** Apelin-13, bioinformatics analysis, early growth response 1, ferritinophagy, ferroptosis, GSE139220, hub gene, nuclear receptor coactivator 4, perioperative neurocognitive disorder, sevoflurane

## Abstract

Sevoflurane is a widely used inhaled anesthetic for inducing and maintaining general anesthesia in surgical patients. After inhalation, sevoflurane crosses the alveolar capillary membrane barrier to enter the circulatory system and act on the central nervous system, producing anesthetic effects. Recent research has found that sevoflurane can induce ferroptosis and trigger neurological disorders. However, the exact process behind this is not yet understood. In this study, we used an integrated approach combining bioinformatics analysis with *in vivo* experiments to investigate the complex relationships between sevoflurane, ferroptosis, and ferritinophagy, along with their underlying mechanisms. By analyzing the sevoflurane anesthesia dataset (GSE139220) in the Gene Expression Omnibus database, we identified the Apelin/early growth response 1 axis as a potential ferroptosis-associated signaling pathway involved in sevoflurane-induced postoperative cognitive dysfunction. The results of *in vivo* experiment showed that sevoflurane could induce perioperative neurocognitive disorder by reducing the ferroptosis markers glutathione peroxidase 4, xCT, and early growth response 1, as well as increasing the ferritinophagy marker nuclear receptor coactivator 4. Importantly, Apelin-13, a subtype of apelin, reversed these effects. Therefore, we concluded that sevoflurane induces ferritinophagy by regulating the apelin/early growth response 1 axis, thereby exacerbating ferroptosis and inducing cognitive impairment. Apelin-13 may be a promising drug for inhibiting sevoflurane-induced perioperative neurocognitive disorder.

## Graphical abstract

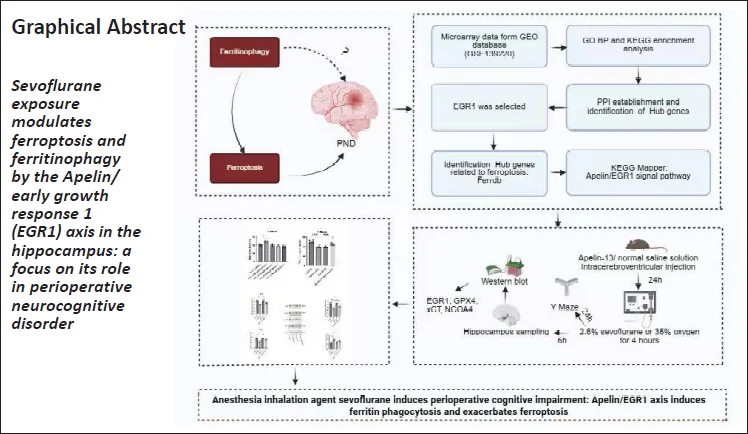

## Introduction

Perioperative neurocognitive disorder is one of the most common postoperative complications, which has a harmful impact on patients’ self-care ability, resulting in longer hospital stays, higher medical costs, morbidity, and mortality among older adults.^1^,^2^ Clinical evidence indicates that up to 60% of patients over 60 years experience cognitive decline following anesthesia exposure, substantially prolonging hospital stays and increasing healthcare costs.^3^,^4^ Sevoflurane, a commonly used general anesthetic, can induce sevoflurane-related perioperative neurocognitive disorder.^5^ The pathogenesis of sevoflurane-induced perioperative neurocognitive disorder appears to involve complex mechanisms, including neuroinflammation, alterations in neurotransmitters, a decrease in brain-derived neurotrophic factor, mitochondrial oxidative stress, and changes in amyloid-β concentrations.^2^

Ferroptosis is a newly recognized form of cell death triggered by lipid peroxide accumulation and iron overload.^6^,^7^ It has been associated with the development of perioperative neurocognitive disorder.^8^,^9^ Evidence suggests that sevoflurane induces neuronal ferroptosis by activating Hippo signaling pathway through the upregulation of protein kinase c δ and perilipin 4.^10^,^11^ The ferroptosis inhibitor liproxstatin-1 alleviates the ferroptosis in aged mice with cognitive deficits induced by sevoflurane anesthesia.^12^ The mechanism of ferroptosis is not fully understood, but the metabolic pathways of iron, lipids, and amino acids are widely recognized as the main ones.^13^ Wu et al.^9^ confirmed that general anesthetics disrupt the balance of iron metabolism in hippocampal neurons by N-methyl-D-aspartate receptor/radical S-adenosyl methionine domain containing 1/divalent metal transporter 1 signaling pathway. The system Xc-/glutathione (GSH)/glutathione peroxidase 4 (GPX4) regulatory axis is an important pathway that negatively regulates ferroptosis. A decrease in GSH-dependent antioxidant defense may be an essential trigger of ferroptosis.^14^,^15^ The system Xc- takes up cystine and exports glutamate via the solute carrier family 7 member 11 (SLC7A11, xCT) subunit. Cysteine can then be used as a raw material for GSH synthesis.^16^ GPX4 inhibits lipid peroxides by converting GSH to oxidized GSH and reducing cytotoxic polyunsaturated fatty acid phospholipid hydroperoxides to non‐cytotoxic polyunsaturated fatty acids phospholipid alcohols during ferroptosis.^17^ SLC7A11 and GSH levels in the hippocampus decreased significantly after isoflurane exposure.^18^ Similarly, lipid reactive oxygen species accumulation is a hallmark of ferroptosis. Exposure to isoflurane has been shown to significantly increase reactive oxygen species levels.^19^ Together, these findings suggest that sevoflurane may contribute to perioperative neurocognitive disorder by promoting ferroptosis, which is induced by an imbalance of iron, amino acids, and lipids.

Ferritinophagy, a process of autophagic degradation of ferritin, has emerged as a pivotal focus in ferroptosis research. It is triggered by the binding of the autophagy receptor protein nuclear receptor coactivator 4 (NCOA4) to ferritin.^20^ NCOA4 then transports the ferritin to the autolysosome for degradation. After ferritin degrades through autophagy, iron is released. This release promotes the expansion of the labile iron pool, which in turn drives the Fenton reaction (Fe^2^ + H_2_O_2_ → Fe^3+^ + ·OH + OH^−^) and causes lipid peroxidation and ferroptosis.^21^ Thus, ferritinophagy also serves as a regulatory mechanism for iron metabolism. When the free iron within cells is insufficient to support life activities, cells initiate ferritinophagy to maintain iron homeostasis. However, when iron ions accumulate excessively within the cell, they generate a large amount of reactive oxygen species via the Fenton reaction, thus inducing ferroptosis. Previous studies have shown that ferritinophagy is, to some extent, the basis of the pathophysiology of neurodegeneration.^22^,^23^ In the propofol-induced cognitive impairment model, decreased GPX4 (a ferroptosis marker) and increased NCOA4 (a ferritinophagy marker), were observed in the hippocampus of the propofol group.^24^ These findings indicate that both ferritinophagy and ferroptosis play a role in this process. However, few reports exist on whether sevoflurane can induce ferritinophagy in the hippocampus. To clarify the roles of ferroptosis and ferritinophagy under sevoflurane exposure, we conducted a bioinformatics analysis and performed animal experiments. Specifically, we used bioinformatics approaches to identify the Apelin/early growth response 1 (EGR1) pathway, which is associated with ferroptosis-linked perioperative neurocognitive disorder. In subsequent animal experiments, we validated this pathway using Apelin-13, a subtype of apelin that offers significant advantages for studying the pathogenesis of neurodegenerative diseases.^25^ Together, our findings suggest a novel target associated with ferroptosis and ferritinophagy for elucidating the mechanism of perioperative neurocognitive disorder and developing therapeutic strategies-Apelin-13/EGR1 axis.

## Methods

### Bioinformation analysis

#### Identification of the differentially expressed genes and functional enrichment analysis

The flow diagram of this study is shown in **Figure 1**. We firstly downloaded the GSE139220 dataset about sevoflurane-anesthetized rats from Gene Expression Omnibus (GEO) database (http://www.ncbi.nlm.nih.gov/geo).^26^ The differentially expressed genes (DEGs) were detected using the Bioconductor linear models (limma) package in the R software (Version 4.3.2, R Foundation for Statistical Computing, Vienna, Austria).^27^ DEGs were identified based on *P* value < 0.05 and |log fold change| ≥ 1. Next, we performed Gene Ontology biology process and Kyoto Encyclopedia of Genes and Genomes (KEGG) enrichment analysis using the clusterProfiler R package.^28^

**Figure 1:**
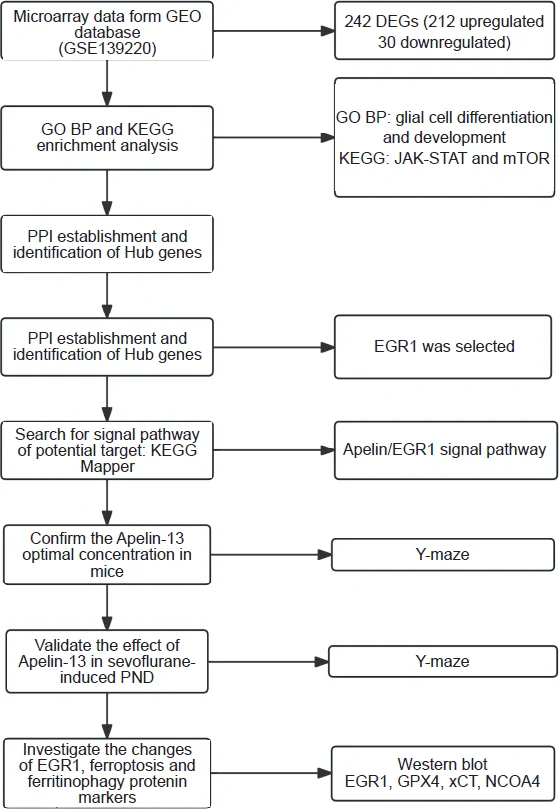
The flowchart of the study procedures. First, the GSE139220 dataset was retrieved to identify differentially expressed genes; subsequently, enrichment analyses and PPI network construction were performed to identify the hub gene EGR1, which is linked to ferroptosis. Next, the Apelin/EGR1 pathway was predicted. Then, the optimal concentration of Apelin-13 was determined using the Y-maze test. Finally, the effect of Apelin-13 on sevoflurane-induced PND was validated by detecting EGR1, ferroptosis, and ferroptoinophagy markers using Western blot. DEGs: Differentially regulated genes; EGR1: early growth response 1; GO BP: Gene Ontology biological process; GPX4: glutathione peroxidase 4; KEGG: Kyoto Encyclopedia of Genes and Genomes; NCOA4: nuclear receptor coactivator 4; PND: perioperative neurocognitive disorders; PPI: protein-protein interaction.

#### Establishment of the protein-protein interaction network and selection of hub genes

The Search Tool for the Retrieval of Interacting Genes/Proteins (STRING) database (https://string-db.org/) was used to analyze protein interactions.^29^ The Cytoscape V3.9.1 (https://cytoscape.org/) was then used to visualize the protein-protein interaction (PPI) network,^30^ which can detect hub genes using the cytoHubba plugin.^30^,^31^ The top 40 nodes were selected.

#### Exploration of hub genes and identification of signal pathway

We downloaded the ferroptosis driver dataset from the Ferrdb V2 database (http://www.zhounan.org/ferrdb/current/)^32^ and explored the intersection of ferroptosis and hub genes using the ggvenn R package. The cell death-related signaling pathway of the selected potential target was mapped using KEGG Mapper (https://www.genome.jp/kegg/)^33^ after searching and conducting an in-depth analysis of a large number of references.

### Validation in mice

#### Animals

Male C57BL/6J mice (aged 6–8 weeks and weighting 20–30 g) were purchased from GemPharmatech (Beijing, China; license No. SCXK (Jing) 2023-0008). They were raised in clean cages under pathogen-free conditions (22–26°C, 12/12-hour light/dark cycle) and had free access to food and water. Mice were adapted to the environment for 1 week before the experiment. After the experimental procedures were completed, all mice were euthanized by cervical dislocation after being anesthetized with sevoflurane (3% in oxygen, flow rate 3 L/min) to ensure minimal discomfort during the procedure. All animal-related experiment procedures strictly complied with the guidelines of the Animal Welfare and Ethics Committee of the Chinese People’s Liberation Army General Hospital (approval No. 2020-X16-75).

#### Experimental design

We hypothesized that Apelin plays a protective role in perioperative neurocognitive disorder by promoting EGR1 expression to inhibit ferritinophagy and ferroptosis as determined by bioinformatics analysis. To validate our hypothesis, we designed animal experiments in three parts (**Figure 2**).

**Figure 2:**
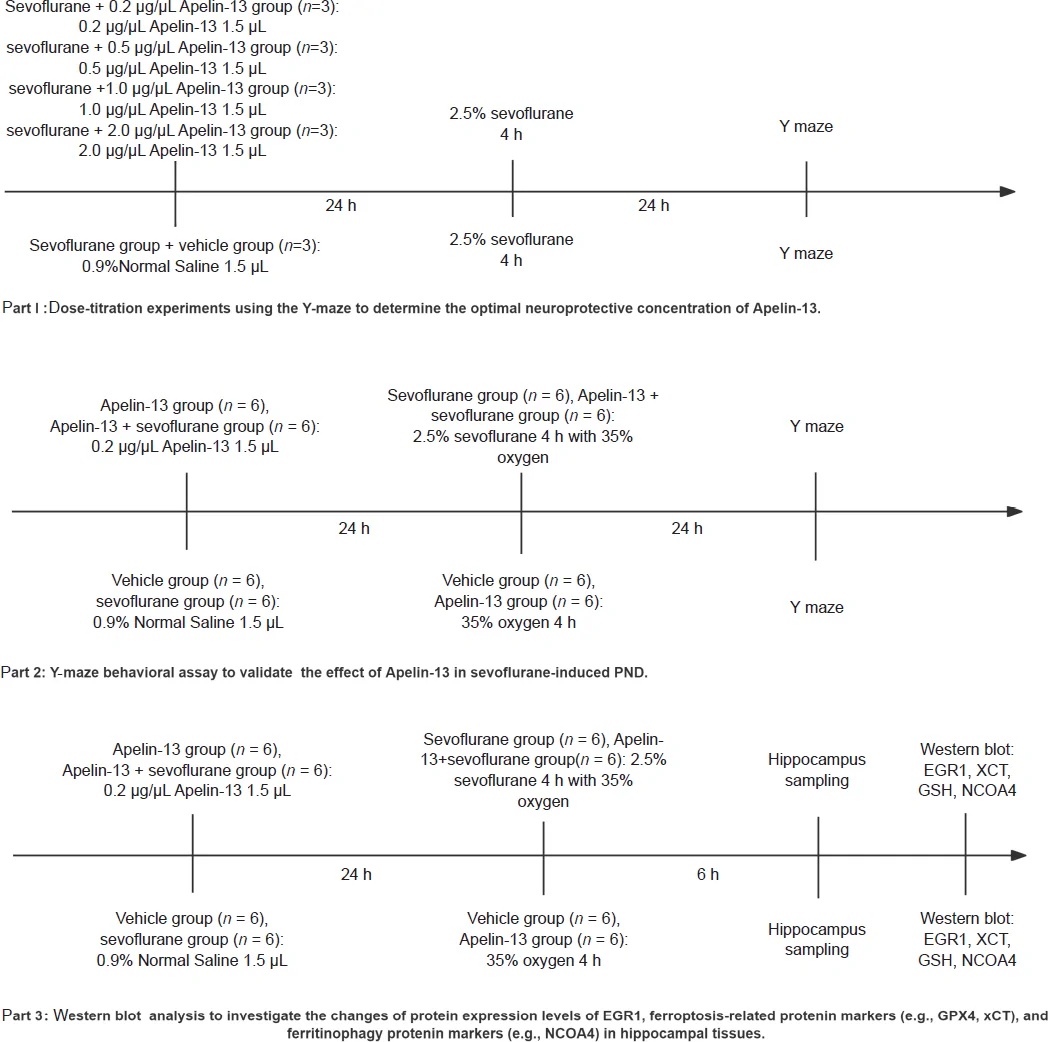
Animal experimental design. EGR1: Early growth response 1; GPX4: glutathione peroxidase 4; NCOA4: nuclear receptor coactivator 4; PND: perioperative neurocognitive disorders.

Part 1: To determine the optimal neuroprotective concentration of Apelin-13, 15 mice were randomly assigned to sevoflurane + vehicle group (*n* = 3), sevoflurane + 0.2 μg/μL Apelin-13 group (*n* = 3), sevoflurane + 0.5 μg/μL Apelin-13 group (*n* = 3), sevoflurane + 1.0 μg/μL Apelin-13 group (*n* = 3), and sevoflurane + 2.0 μg/μL Apelin-13 group (*n* = 3). Apelin-13 (217082-58-1, Solarbio, Beijing, China) was dissolved in pyrogen-free 0.9% normal saline. For the hippocampus injection of 1.5 μL of Apelin-13 solution (0.2 μg/μL) or normal saline, the mice were placed on a stereotactic apparatus (Zhongshi Dichuang Technology, Beijing, China) after being intraperitoneally anesthetized with sodium pentobarbital (40 mg/kg; Hubei Hongyun Long Biological Technology Co. Ltd., Wuhan, China). Using the anterior fontanelle as the reference point, the injection coordinates were set to posterior: 0.5 mm, lateral: ±1.0 mm, and ventral: 2.0 mm to target the hippocampus.^34^ Apelin-13 or normal saline was injected at a uniform speed within 3 minutes, and the needle was left in place for at least 3 minutes after injection. All groups received 2.5% sevoflurane (24081231, Shanghai Hengrui, Shanghai, China) in 35% oxygen for 4 hours in an anesthetizing chamber. Twenty-four hours after sevoflurane anesthesia, the mice underwent a Y-maze behavioral assessment to evaluate cognitive function. The Apelin-13 concentration and the behavioral assessment time were set according to previous studies.^35^,^36^

Part 2: We validated the potential causal link between sevoflurane exposure and perioperative neurocognitive disorder pathogenesis, as well as the protective effect of Apelin-13 against sevoflurane-induced cognitive deficits. Twenty-four mice were randomly assigned to vehicle group (*n* = 6), sevoflurane group (*n* = 6), Apelin-13 group (*n* = 6) and Apelin-13 + sevoflurane group (*n* = 6). Mice in the vehicle and sevoflurane groups received 1.5 μL of normal saline in their hippocampus, while the mice in Apelin-13 and Apelin-13 + sevoflurane groups were received 1.5 μL of Apelin-13 (0.2 μg/μL). Twenty-four hours after receiving sevoflurane anesthesia or 35% oxygen, all mice underwent Y-maze behavioral testing.

Part 3: To determine whether sevoflurane could induce alterations in the expression levels of EGR1, ferroptosis markers (e.g., GPX4 and xCT), and ferritinophagy markers (e.g., NCOA4) in hippocampal tissues, we investigated changes in these proteins. We also examined whether Apelin-13 could reverse these changes after confirming the optimal Apelin-13 concentration. Mice were randomly assigned to vehicle group (*n* = 6), sevoflurane group (*n* = 6), Apelin-13 group (*n* = 6) and Apelin-13 + sevoflurane group (*n* = 6). The procedure was the same as in Part 2. Twenty-four hours after injection, vehicle and Apelin-13 groups received 35% oxygen at 4 L/min for 4 hours in an identical chamber. Meanwhile, sevoflurane and Apelin-13 + sevoflurane groups received 2.5% sevoflurane in 35% oxygen for 4 hours in an anesthetizing chamber. Six hours later, the mice were euthanized, and their hippocampal tissues were collected. The protein expression of EGR1, NCOA4, GPX4, and xCT was detected by Western blot assay.

#### Y-maze test

The novel arm test in the Y-maze test was used to evaluate the cognitive function.^37^ The Y-maze apparatus (Zhongshi Dichuang Technology, Beijing, China) has three identical arms labeled A, B, and C, which are arranged at 120° angles. Each arm measures 40 cm in length, 10 cm in width, and 15 cm in height. The maze is made of an opaque material that blocks external visual cues. Testing was conducted during the light phase (between 9:00 a.m. and 12:00 p.m.). Each mouse was placed at the end of one designated arm. The testing included three phases: habituation, training and testing. During the habituation phase, the animals were placed in one of the arms of the Y-maze and allowed to freely explore all three arms for 5 minutes. After the habituation phase, the animals were reintroduced to the maze and allowed to freely explore the accessible arms for 5 minutes. Exploratory behaviors were documented using a video-tracking system (Stoelting, Kiel, WI, USA). Then, the animals were promptly returned to their housing facility. During the testing phase, the partition was removed to open all three arms. The animal was placed in the maze facing away from the arms, and its exploratory behaviors were recorded for 5 minutes. An arm entry was recorded only when all four of the mouse’s paws entered the arm. The following parameters were recorded: each arm entry, each arm duration, and each arm distance. The percentage of time spent in the novel arm was calculated, and the longer the time spent in the novel arm, the better the cognitive function.

#### Western blot analysis

Total protein was extracted from hippocampal tissue using a lysis buffer. After quantification using a bicinchoninic acid protein assay kit (Solarbio), 30 μg of protein samples were loaded onto 4–12% gradient sodium dodecyl sulfate-polyacrylamide gel electrophoresis gel, and then transferred to polyvinylidene difluoride membranes. The membranes were blocked with rapid blocking buffer (Yangguang Bio, Beijing, China) for 1 minute and then incubated with primary antibodies at 4°C overnight. The membranes were subsequently incubated with horseradish peroxidase-linked anti-rabbit antibody (1:10,000, Jackson ImmunoResearch Laboratories, West Grove, PA, USA, Cat# 111-035-003, RRID: AB_228341) for 1.5 hours at room temperature. Next, the protein bands were visualized using an Odyssey scanner (LI-COR, Lincoln, NE, USA). The images were analyzed using ImageJ software (Version 1.54 g, National Institutes of Health, Bethesda, MD, USA). We used β-actin as the loading control (internal reference protein) to normalize the data. The optical density of the target protein bands is measured first, and then the target protein intensity is normalized to the intensity of the corresponding β-Actin bands in the same sample. The primary antibodies were EGR1 (rabbit, 1:1000, Proteintech, Wuhan, China, Cat# 22008-1-AP, RRID:AB_11182923), NCOA4 (rabbit, 1:1000, Proteintech, Cat# 10968-1-AP, RRID: AB_3669123), GPX4 (rabbit, 1:1000, Affinity Bioscience, Nanjing, China, Cat# DF6701, RRID: AB_2838663), xCT (rabbit, 1:1000, MedChemExoress, Princeton, NJ, USA, Cat# HY-P80935, RRID:AB_3102762), and β-actin (rabbit, 1:1000, Affinity Bioscience, Cat# AF7018, RRID:AB_2839420).

### Statistical analysis

All statistical data from the animal experiments were analyzed using GraphPad Prism version 10.1.1 for Windows (GraphPad Software, Boston, MA, USA, www.graphpad.com). The sample size was determined based on our previous study.^35^ All mice completed the entire experimental process. The evaluator was blinded to grouping. Data are expressed as mean ± standard error. Differences among four or five groups were compared using one-way analysis of variance with Bonferroni’s *post hoc* test. A *P*-value of < 0.05 was considered statistically significant.

## Results

### Differentially expressed gene identification induced by sevoflurane anesthesia

The differential expression analysis of GSE139220, compared with the control group, 212 genes were upregulated and 30 genes were downregulated in the sevoflurane group, where animals were exposed to 2.5% sevoflurane in 100% oxygen for 4 hours in an anesthetizing chamber. The result is displayed in **Figure 3** and **Table 1**.

**Figure 3:**
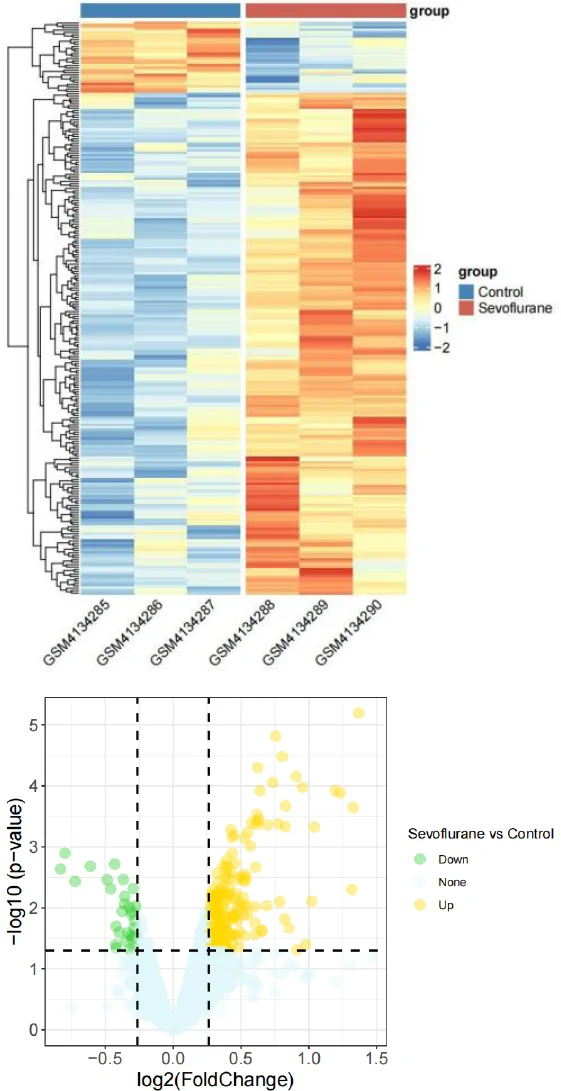
Differentially expressed genes (DEGs) between the control and sevoflurane-treated groups. (A) Heatmap of DEGs. (B) Volcano plot of DEGs. There were 212 upregulated and 30 downregulated genes in the sevoflurane group compared with the control group.

**Table 1:** Differentially regulated genes between the control and sevoflurane groups

	Number	Differentially regulated genes
Upregulated genes	212	*Gpd1, Gjb6, Mt2A, Ret, Mt1m, Hif3a, Sult1a1, Gpatch4, Pnpla2, Prkcd, Pla2g3, Slitrk6, Fgf2, Hmgcs2, Etnppl, Tnfrsf11a, Mfsd2a, Zbtb16, Cdkn1a, Mt1a, Rpe65, Nwd1, Pcp4, Pnpla7, Fn1, berflo, Slc2a1, Olr1581, Slc2a9, Ptpn11, Tsc22d3, Slc38a2, Prr5, Rassf4, Ddit4, Bag3, Acer2, Rora, Usp54, Ptp4a3, Otud7b, Plcd4, O1r455, Cryab, Fzd9, Cnksr3, Arrdc2, Ndrg1, Zfhnx3, Pdk4, Zcchc24, Snx9, Atp6v0e1, Hapln2, Prodh, Spint1, Lims2, Ralgds, Sf3b5, Kcnc2, Plat, Pdlim1, Tfcp2l1, Peli2, Fkbp5, Fzd8, Rgs16, Agtrap, Vom1r27, Tmem140, Tmem252, Slc17a6, Shox2, Aadat, Ednrb, Itgb5, Olr488, Tmem100, Smox, Sult1d1, Rcan2, Slc25a13, Aldoc, Slc19a3, Adipor2, Lonrf3, C1qc, Il10ra, Creg1, Fitm2, Kcnj16, Itga9, Npc1, Mal, Pdgfra, Map3k6, Pex11a, Endod1, Hyal1, Cntfr, Cebpd, Sval2, Mertk, Synpo2, Ccl6, Dusp12, Tppp3, Olr1548, Syngr2, Plin4, Agt, Olr382, Parvg, Klf9, Sult5a1, Rpl18, Mlc1, Olr778, Olr1172, Pygm, Inpp4b, Sgk1, Rab31, Spsb1, Pramef20, Unc119b, Ddah1, Arhgef10, Cygb, Olr1541, Rac2, Cables1, Plcb4, Aox1, Retsat, Gadd45a, Hip1, Tinagl1, Krtap13-2, Sparc, A2m, Dbt, Pip4k2a, Tmem163, Rhob, P3h2, Tcf7l2, Ankrd34b, Enpp4, Dio2, Gramd3, Mag, Omd, Olr1658, Alox5ap, Olr1335, Car6, Tinagl1, Krtap13-2, Sparc, A2m, Dbt, Olr1658, Alox5ap, Olr1335, Car6, Tinagl1, Krtap13-2, Sparc, A2m, Dbt, Pip4k2a, Tmem163, Rhob, P3h2, Tcf7l2, Ankrd34b, Enpp4, Dio2, Gramd3, Mag, Omd, Olr1658, Alox5ap, Olr1335, Car6, Hrk, Mtif3, Eya1, Trim25, Lmod1, Klf15, Cartpt, Rtkn, Errfi1, Olr160, Gpr139, Pter, Tbxas1, Clec12a, Slc10a6, Fgd6, Ehd2, Olig1, Pate4, plorlor, Slc6a11, Gatm, Igsf1, Fam83d, Elovl1, Mgst2, Hand1, Vim, Ctxn2, Timp3, Scn7a, Anlnl1, Olr756, Diras3, C3, Sipa1, Vom1r92, Porf1, Ighv1-12, Olr1407, Acsl3, Abhd15, Serpinb3a, Olr466, Gbp2, Prkg2, Olr425, Cd44, Olr471, Olr346, Aspa, GSTA1, Mettl11b, Gsta1, V-set.20*
Downregulated genes	30	*Itm2a, Slco1a1, Opalin;Lair1, Prom1, Adpgk, Slco1c1, Nr4a3, Hba-a1, Tmc7, Amy1a, Rara, Raph1, Nrarp, Vom1r17, Fzd2, Cacng3, Arc, Tspan2, Egr1, Lgals9, Pdlim3, Abca1, Eef1b2l, Gpam, Kcnv1, Gfral, Spag6, Olr365, Kcnh7*

### Functional enrichment analysis of differentially expressed genes induced by sevoflurane anesthesia

Gene Ontology biology process and KEGG analyses were performed to explore the functional roles of DEGs. Twenty-five biological process terms were significantly enriched, with DEGs showing strong associations with glial cell differentiation and development (**Figure 4**). These processes are closely linked to neuronal death. KEGG pathway analysis revealed that these DEGs were predominantly enriched in the Janus kinase/signal transducer and activator of transcription signaling pathway and mammalian target of rapamycin signaling pathway (**Figure 5**).

**Figure 4:**
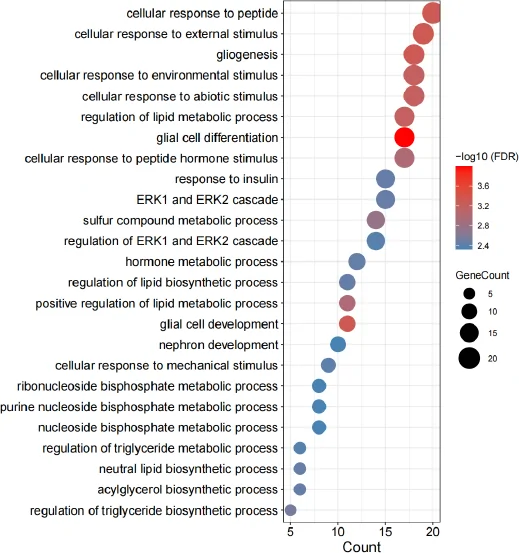
Glial cell differentiation and development are enriched in Gene Ontology biological processes. The red box indicates the enrichment biological process.

**Figure 5:**
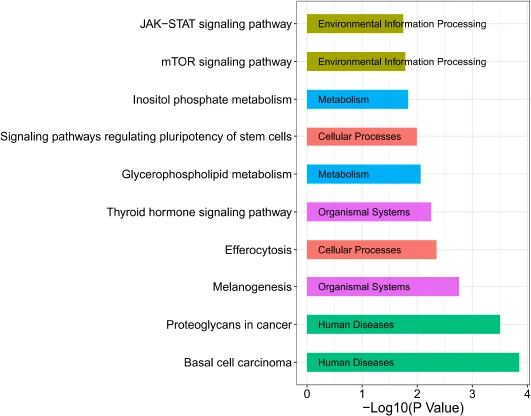
Kyoto Encyclopedia of Genes and Genomes enrichment analysis of differentially expressed genes (DEGs). These DEGs are enriched in the JAK/STAT and mTOR signaling pathways. JAK: Janus kinase; mTOR: mammalian target of rapamycin; STAT: signal transducer and activator of transcription.

### Protein–protein interaction network construction, hub gene selection, and ferroptosis-related hub gene exploration

PPI analysis of DEGs was performed using the STRING database, identifying a network of 133 nodes and 190 interactions. The top 40 hub genes are visualized in **Figure 6**. Among these, two genes (*EGR1* and *NDRG1*) were significantly associated with ferroptosis (**Figure 7**). Based on extensive literature evidence, *EGR1* was selected as the target gene for further analysis. KEGG pathway mapping revealed that the Apelin signaling pathway acts as an upstream regulator of *EGR1*.^38^ Bioinformatics analysis indicates that apelin may inhibit the ferroptosis through *EGR1*, thereby attenuating perioperative neurocognitive disorder.

**Figure 6:**
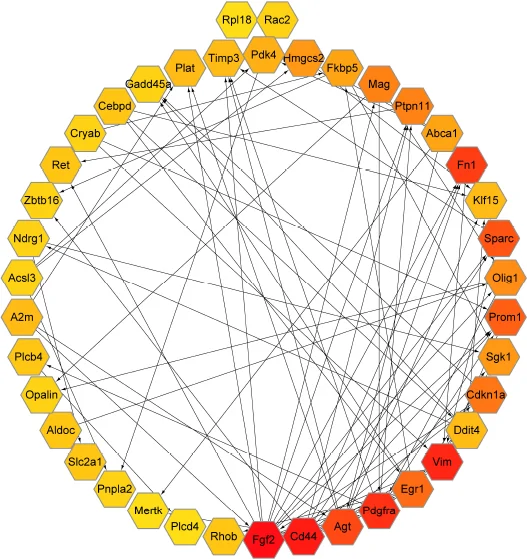
Protein-protein interaction network of differentially expressed genes. The network includes 133 nodes and 190 edges. Each node represents a gene or the protein encoded by that gene. Different colors (yellow, orange, and red) typically indicate different expression levels, in which red might signify highly upregulated genes, while yellow indicates less strongly regulated genes. Edges represent interactions between genes/proteins.

**Figure 7:**
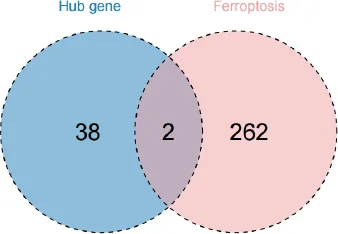
Venn diagram of overlap between Hub genes and ferroptosis-related genes. There are two genes that overlap between hub genes and ferroptosis-related genes.

### Sevoflurane-induced perioperative neurocognitive disorder is ameliorated by Apelin-13

In the Apelin-13 concentration experiment (part 1), we found that the optimal concentration was 0.2 μg/μL (*P* < 0.05). Furthermore, compared with the vehicle group, sevoflurane-treated mice exhibited a significantly reduced percentage of time spent in the novel arm in the Y-maze test in part 2 experiment (*P* < 0.001). Notably, the Apelin-13 + sevoflurane group showed a significant increase in time spent exploring the novel arm compared with the sevoflurane group (*P* < 0.001; **Figure 8**). These findings demonstrate that sevoflurane induces perioperative neurocognitive disorder, whereas Apelin-13 effectively ameliorates sevoflurane-associated cognitive impairment.

**Figure 8:**
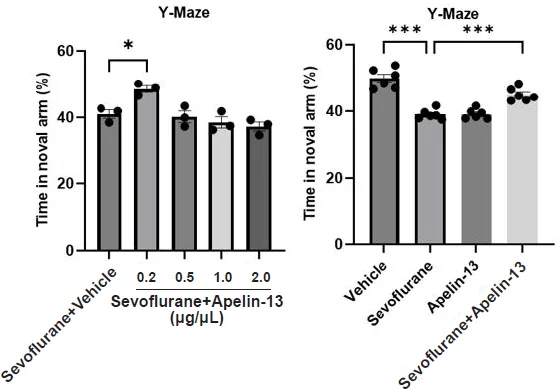
Apelin-13 ameliorates sevoflurane-induced perioperative neurocognitive disorder as detected by the novel arm of the Y-maze test. Data are expressed as mean ± SEM. ^*^P < 0.05, ^***^P < 0.001 (one-way analysis of variance with Bonferroni’s *post hoc* test).

### Sevoflurane-induced decrease in early growth response 1 is reversed by Apelin-13

Compared to the vehicle group, the results of western blot assay (part 3) showed that EGR1 expression was significantly reduced in the sevoflurane group (*P* < 0.001). In contrast, the Apelin-13 + sevoflurane group exhibited higher EGR1 levels than the sevoflurane group (*P* < 0.01, **Figure 9**). These findings indicate that sevoflurane suppresses EGR1 expression, while Apelin-13 rescues this suppression.

**Figure 9:**
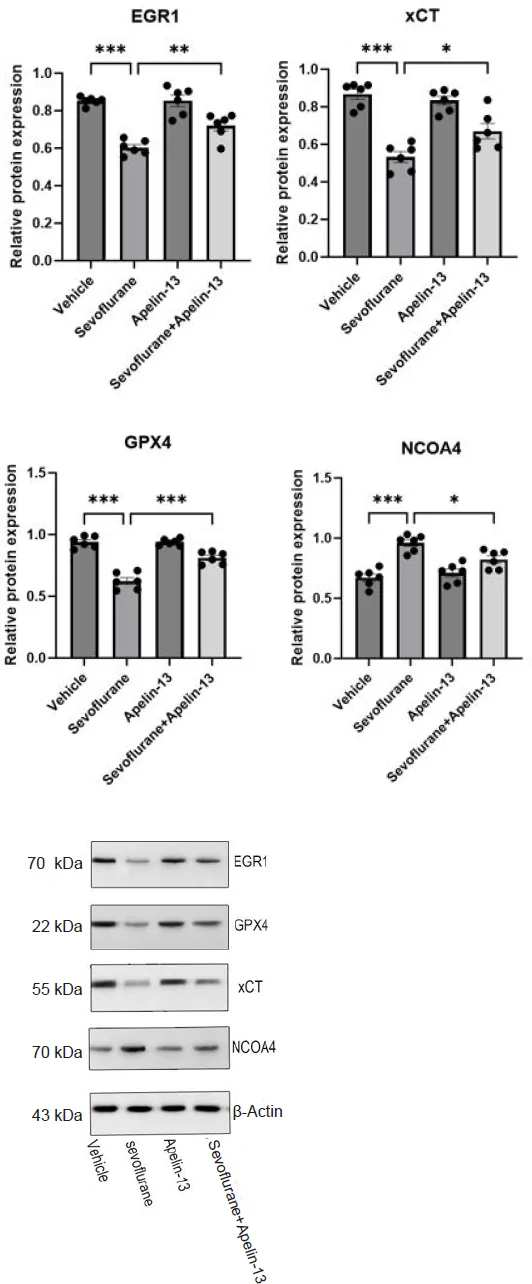
Apelin-13 ameliorates sevoflurane-induced EGR1, GPX4, xCT, and NCOA4 expression change as detected by western blot analysis. Data are normalized by β-actin are expressed as mean ± SEM. ^*^P < 0.05, ^**^P < 0.01, ^***^P < 0.001 (one-way analysis of variance with Bonferroni’s *post hoc* test). EGR1: Early growth response 1; GPX4: glutathione peroxidase 4; NCOA4: nuclear receptor co-activator 4; xCT: solute carrier family 7 member 11.

### Sevoflurane-induced ferroptosis and ferritinophagy are mitigated by Apelin-13

As depicted in **Figure 9**, the results of western blot (part 3) indicated that the expression levels of GPX4 and xCT in the sevoflurane group were significantly lower than those in the vehicle group (*P* < 0.001). Conversely, the Apelin-13 + sevoflurane group exhibited increased levels of GPX4 and xCT compared to the sevoflurane group (*P* < 0.001). These results demonstrate that sevoflurane induces ferroptosis in the mouse hippocampus, while Apelin-13 effectively inhibits this process.

Western blot analysis revealed that NCOA4 levels, a ferritinophagy biomarker,^24^ were significantly higher in the sevoflurane group than in the vehicle group (*P* < 0.001). However, NCOA4 levels were markedly reduced in the Apelin-13 + sevoflurane group compared with sevoflurane group (*P* < 0.01; **Figure 9**). These results suggest that sevoflurane promotes the initiation and progression of ferritinophagy, whereas Apelin-13 exerts a suppressive effect.

Overall, the results demonstrated that sevoflurane induces ferroptosis and ferritinophagy and EGR1 downregulation, thereby leading to perioperative neurocognitive disorder. Notably, Apelin-13 effectively inhibits sevoflurane-induced ferroptosis and ferritinophagy.

## Discussion

Our findings demonstrate that sevoflurane promotes ferroptosis and ferritinophagy by suppressing EGR1, thereby contributing to perioperative neurocognitive disorder, and these effects are reversed by Apelin-13. For the first time, we establish a link between sevoflurane exposure, EGR1 downregulation, and ferroptosis and ferritinophagy in perioperative neurocognitive disorder. Additionally, we identified Apelin-13 as a potential agent for preventing sevoflurane-induced neurotoxicity due to its ability to restore EGR1 expression and inhibit ferroptosis and ferritinophagy. Our results provide a new perspective on the mechanisms of perioperative neurocognitive disorders and identify potential therapeutic targets.

Our study further validated the widely accepted notion that sevoflurane can induce perioperative neurocognitive disorder. We observed a significant reduction in EGR1 expression following sevoflurane exposure, which highlights the critical role of EGR1 in this context. EGR1, also known as Zif268 or Krox-24, acts as a transcription factor and is classified as an “early gene” that can be quickly activated in response to various stimuli.^39^ Recent research has increasingly focused on the association between sevoflurane-induced perioperative neurocognitive disorder and EGR1. Within the hippocampus, EGR1 expression remains relatively elevated during normal sustained neuronal activity,^40^ peaking at 30 to 60 minutes following stress and declining significantly by 120 minutes post-stress.^41^ A previous study has shown that sevoflurane inhibits EGR1 expression during and after anesthesia.^42^ This suppression persists for at least 24 hours after anesthesia. Our findings corroborate this conclusion. As we have emphasized, neuroinflammation is one of the underlying mechanisms of sevoflurane-induced perioperative neurocognitive disorder. Research indicates a strong association between EGR1 and neuroinflammation.^43^ Therefore, it is plausible that sevoflurane may induce neuroinflammation and contribute to perioperative neurocognitive disorder through its effects on EGR1. However, there is an ongoing debate regarding the modulation of EGR1 levels in the context of neuroinflammation. A study involving rats with lipopolysaccharide-induced neuroinflammation has reported a reduction in EGR1 expression.^44^ Similarly, a decrease in *EGR1* gene expression was observed in a type 2 diabetes mellitus mouse model.^45^ However, Yu et al.^46^ identified EGR1 activation as a promoter of neuroinflammation. This discrepancy may arise from various factors, necessitating further investigation to elucidate the underlying causes.

Our study also demonstrated that sevoflurane induces ferroptosis and ferritinophagy in the mouse hippocampus. Previous research has shown that sevoflurane can trigger ferroptosis by affecting iron metabolism.^8^ Our findings on the impact of sevoflurane on ferroptosis-related proteins align with this, showing its significant regulatory role in iron metabolism and the ferroptosis pathway. Beyond its association with sevoflurane, EGR1 is also implicated in ferroptosis. Ding et al.^47^ were the first to reveal that GPX4 regulates mitochondria-mediated apoptosis through EGR1 modulation in triple-negative breast cancer cells. In our experiments, we observed alterations in both EGR1 and ferroptosis concurrently. Consequently, we hypothesize that EGR1 may be involved in sevoflurane-induced ferroptosis, which requires further experimental validation.

Our findings suggest that sevoflurane administration results in the upregulation of ferritinophagy-related proteins, specifically NCOA4. This indicates that sevoflurane can induce ferritinophagy. However, the underlying mechanisms of this process are not well understood, and more research is needed.

Apelin, the endogenous ligand for the G-protein-coupled receptor APJ,^48^ has demonstrated neuroprotective effects and the ability to mitigate neuronal apoptosis,^49^ thereby playing a significant role in cognitive dysfunction.^50^ Apelin can be categorized into several subtypes, including Apelin-13, Apelin-17, and Apelin-36, which differ in their amino acid structures.^51^ Consistent with previous studies on neuropeptide neuroprotection, Apelin-13 effectively ameliorated sevoflurane-induced cognitive decline, highlighting its potential clinical applications in anesthesia. Apelin-13 has also been reported to improve cognitive deficits and promote the recovery of hippocampal neuronal damage by activating peroxisome proliferator-activated receptor-γ coactivator-1α and peroxisome proliferator-activated receptor-γ signaling.^51^ Similarly, Apelin-13 has been demonstrated to exert neuroprotective effects in Alzheimer’s disease.^35^

However, the relationship between Apelin-13 and sevoflurane-induced ferroptosis and ferritinophagy has been scarcely explored in prior research. Our study provides preliminary insights into the role of Apelin-13 in these pathological processes, suggesting that Apelin-13 can attenuate their progression. Additionally, our findings indicate that Apelin-13 may exert its effects through EGR1 activation. Nevertheless, further research is necessary to elucidate the underlying mechanisms.

This study has several limitations that must be acknowledged. First, the conclusions were drawn primarily using Western blot analysis and the Y-maze test rather than a range of experimental methodologies, which may affect the comprehensiveness and reliability of the findings. Furthermore, in future studies, it would be beneficial to consider expanding the sample sizes of animal groups for behavioral analyses, while incorporating additional behavioral testing methods, such as the water maze test, to facilitate a more comprehensive cognitive evaluation. Second, the molecular mechanisms linking EGR1 to ferroptosis and ferritinophagy are unclear, necessitating targeted mechanistic studies using EGR1 genetic knockout models. Third, although Apelin-13 has been shown to reverse the decrease in EGR1 and mitigate ferroptosis and ferritinophagy, the precise mechanisms involved remain unclear. Apelin-13 probably ameliorates sevoflurane-induced perioperative neurocognitive disorder, inhibiting ferroptosis and ferritinophagy in the hippocampus through EGR1. Fourth, the experiments were conducted exclusively in mice and lack validation in human subjects or cell models. Our experiment was mainly based on the findings from a previous study^35^ that male animals exhibit more stable cognitive impairment responses to sevoflurane exposure than females and that this eliminates the interference of periodic fluctuations in sex hormones on experimental results. However, this design also limits the gender representativeness of the study conclusions. An existing study has shown that estrogen in female animals can regulate the key ferroptosis-related molecules (e.g., GPX4), thereby affecting the activity of the ferritinophagy-ferroptosis axis.^52^ Therefore, future studies should include experiments with female animals to verify the regulatory effect of sex hormones on sevoflurane-induced perioperative neurocognitive disorder. This will enhance the comprehensiveness of the research conclusions and their clinical translational value.

In conclusion, sevoflurane has been shown to induce ferroptosis and ferritinophagy in the hippocampus, probably by inhibiting EGR1, thereby leading to perioperative neurocognitive disorder. Conversely, Apelin-13 has been found to reverse the reduction in EGR1 expression, alleviating sevoflurane-induced ferroptosis and ferritinophagy in mice and improving perioperative neurocognitive disorder. These findings improve our understanding of the roles of EGR1, ferroptosis, and ferritinophagy in sevoflurane exposure. Furthermore, they provide evidence that supports developing Apelin-13 as a therapeutic agent to prevent perioperative neurocognitive disorder. However, more research is needed to address these limitations and further elucidate the mechanisms involved.

## Data Availability

*The datasets of this article are available from the corresponding author upon reasonable request.*
